# FGF21 deletion exacerbates diabetic cardiomyopathy by aggravating cardiac lipid accumulation

**DOI:** 10.1111/jcmm.12530

**Published:** 2015-03-30

**Authors:** Xiaoqing Yan, Jun Chen, Chi Zhang, Shanshan Zhou, Zhiguo Zhang, Jing Chen, Wenke Feng, Xiaokun Li, Yi Tan

**Affiliations:** a Chinese-American Research Institute for Diabetic Complications at the Wenzhou Medical UniversityWenzhou, China; b Kosair Children’s Hospital Research Institute, The Department of Pediatrics of the University of Louisville, School of MedicineLouisville, USA; c Scool of Nursing, Wenzhou Medical UniversityWenzhou, China; d Department of Cardiovascular Disorders of the First Hospital of Jilin UniversityChangchun, China; e Department of Pharmacology and Toxicology of the University of Louisville School of MedicineLouisville, USA

**Keywords:** fibroblast growth factor 21, diabetic cardiomyopathy, CD36, cardiac lipotoxicity

## Abstract

Fibroblast growth factor 21 (FGF21) plays an important role in energy homoeostasis. The unaddressed question of FGF21’s effect on the development and progression of diabetic cardiomyopathy (DCM) is investigated here with FGF21 knockout (FGF21KO) diabetic mice. Type 1 diabetes was induced in both FGF21KO and C57BL/6J wild-type (WT) mice *via* streptozotocin. At 1, 2 and 4 months after diabetes onset, the plasma FGF21 levels were significantly decreased in WT diabetic mice compared to controls. There was no significant difference between FGF21KO and WT diabetic mice in blood glucose and triglyceride levels. FGF21KO diabetic mice showed earlier and more severe cardiac dysfunction, remodelling and oxidative stress, as well as greater increase in cardiac lipid accumulation than WT diabetic mice. Western blots showed that increased cardiac lipid accumulation was accompanied by further increases in the expression of nuclear factor (erythroid-derived 2)-like 2 (Nrf2) and its target protein CD36, along with decreases in the phosphorylation of AMP-activated protein kinase and the expression of hexokinase II and peroxisome proliferator-activated receptor gamma co-activator 1α in the heart of FGF21KO diabetic mice compared to WT diabetic mice. Our results demonstrate that FGF21 deletion-aggravated cardiac lipid accumulation is likely mediated by cardiac Nrf2-driven CD36 up-regulation, which may contribute to the increased cardiac oxidative stress and remodelling, and the eventual development of DCM. These findings suggest that FGF21 may be a therapeutic target for the treatment of DCM.

## Introduction

Diabetic cardiomyopathy (DCM) is one of the most severe diabetic complications. It has been defined as ventricular dysfunction that occurs independently of coronary artery disease and hypertension 1. The pathogenesis of DCM is multifactorial 2. Altered myocardial substrate and energy metabolism has emerged as an important contributor to the development of DCM 3. Despite an increase in fatty acid use in diabetic hearts, fatty acid uptake likely exceeds its oxidation rate, thereby resulting in cardiac lipid accumulation that promotes cardiac lipotoxicity 4. Thus, targeting to correct diabetes-induced abnormal substrate metabolism in the heart will potentially lower the prevalence of DCM, thereby improve long-term survival of the patients with diabetes.

Fibroblast growth factor 21 (FGF21), a novel member of the FGF family, encoded by the *fgf21* gene located in chromosome 19 in human 5 and chromosome 7 in mice, has been identified as a potent metabolic regulator with specific effects on glucose and lipid metabolism 6. FGF21 is preferentially expressed in the liver 5. But other tissues, such as pancreas 7, white 8 and brown 9 adipose tissues, skeletal muscle 10 and heart 7 also express FGF21. FGF21 stimulates glucose uptake in adipocytes *via* the induction of glucose transporter-1, which is additive and independent of insulin 11. Under hypothermic conditions, FGF21 can induce browning of white adipose tissues to up-regulate thermogenic activity, which could, at least in part, lead to a greater clearance of glucose 12. Moreover, FGF21 has shown beneficial effects on lipid profiles in animal models 13,14. FGF21 can also regulate lipolysis in adipocytes in response to fasting 15. Treatment with FGF21 enhances the expression and secretion of downstream effector adiponectin in adipocytes which in turn further improves fatty acid oxidation and lipid clearance in the liver and skeletal muscle 16.

Since its benefits in regulating glucose and lipid metabolism, FGF21 has shown therapeutic potential in treating diabetes 17. FGF21 has insulin-sensitizing ability 16 and can ameliorate glucose tolerance 18 by reducing hepatic glucose production and stimulating glucose uptake in adipocytes. Acute FGF21 treatment suppressed hepatic glucose production, increased liver glycogen, lowered glucagon and improved glucose clearance in *ob/+* mice, while chronic FGF21 treatment ameliorated fasting hyperglycaemia in *ob/ob* mice *via* increased glucose disposal and improved hepatic insulin sensitivity 19. Besides those insulin-mimetic properties, FGF21 does not induce mitogenicity, hypoglycaemia or weight gain at any dose tested in diabetic or healthy animals or when overexpressed in transgenic mice 13; therefore, FGF21 shows a promise as an effective treatment of diabetes.

To date, the function of FGF21 has been extensively investigated, but most studies focused on the liver, adipose tissue 20 and skeletal muscle 10,16. The effect of FGF21 on the heart has been neglected. FGF21 activity depends on its binding to the fibroblast growth factor receptor 1 (FGFR1), especially FGFR1c, and co-factor β-Klotho 21. The existence of FGFR1c, β-Klotho 22 and FGF21 7 in the heart suggests that FGF21 may play certain roles in the physiological and pathophysiological aspects of the heart. Recently, FGF21 was found to have cardio-protective effects against myocardial ischaemia/reperfusion injury 23 and isoproterenol-induced cardiac hypertrophy 24. However, its effect on DCM has not been characterized. In this study, we investigated the effect of FGF21 deletion on the development of DCM in a FGF21 knockout (FGF21KO) mouse model. We found that mice lacking the *fgf21* gene are more prone to develop DCM, which is likely because of the overexpression of CD36-mediated cardiac lipid accumulation.

## Materials and methods

### Ethics statement

This study was carried out in strict accordance with the recommendations in the Guide for the Care and Use of Laboratory Animals of the National Institutes of Health. The protocol was approved by the Animal Policy and Welfare Committee of Wenzhou Medical University and the Institutional Animal Care and Use Committee of the University of Louisville. All surgeries were performed under anaesthesia induced by intraperitoneal injection of 1.2% 2,2,2-Tribromoethanol (avertin) at the dose of 300 mg/kg bw and all efforts were made to minimize suffering.

### Animal model

Male FGF21KO mice with C57 BL/6J background (gift from Dr. Steve Kliewer, University of Texas Southwestern Medical Center) and wild-type (WT) C57 BL/6J mice purchased from Jackson Laboratory (Bar Harbor, ME, USA) were used in this study. Type 1 diabetic mouse model was induced in 10 week-old male FGF21KO mice and age-matched WT mice by intraperitoneal (i.p.) injection of six doses of streptozotocin (STZ, Sigma-Aldrich, St. Louis, MO, USA in 10 mM sodium citrate buffer, pH 4.5) at 60 mg/kg bw daily. Control group (Ctrl) of FGF21KO and WT mice received citrate buffer alone. Seven days after the last STZ injection, whole blood glucose obtained from the mouse tail vein was detected using a SureStep complete blood glucose monitor (LifeScan, Milpitas, CA, USA), and animals with blood glucose levels greater than 250 mg/dl were considered diabetic. At 1, 2 and 4 months after diabetes onset, heart function and blood pressure were measured and mice were then killed.

### Echocardiography

At 1, 2 and 4 months after diabetes onset, heart function was evaluated by transthoracic echocardiography (ECHO). ECHO was performed on mice using a Visual Sonics Vevo 770 high-resolution imaging system (Visual Sonics, Toronto, ON, Canada) and equipped with a RMV 707B High-Frame-Rate Scanhead (focal length 12.7 mm, frequency 30.0 MHz), as described previously 25. Under sedation with avertin (300 mg/kg bw), mice were placed in a supine position on a heating pad to maintain body temperature at 36–37°C that was continuously monitored using a rectal thermometer probe. Under these conditions, the animal’s heart rate ranged between 400 and 550 beats/min. Two-dimensional and M-mode ECHO were used to assess wall motion, chamber dimensions and cardiac function.

### Blood pressure measurement

Blood pressure was measured using a CODA™ mouse/rat tail-cuff system (Kent scientific, Torrington, CT, USA) following our previous published protocol 25.

### Plasma FGF21 assay

Whole blood was collected in a lithium heparin tube (BD, Franklin Lakes, NJ, USA), and centrifuged at 2000 r.p.m. for 20 min. Then, plasma was collected for FGF21 assay using a FGF21 Quantikine Elisa kit (R&D systems, Minneapolis, MN, USA) according to the manufacturer’s instructions.

### Plasma and cardiac triglyceride assay

Triglyceride concentrations were measured using a triglyceride assay kit (Cayman Chemicals, Ann Arbor, MI, USA) according to the manufacture’s protocol. For plasma triglyceride assay, plasma from diabetic mice was diluted 1:1 with standard diluent assay reagent, while the plasma from control mice was not diluted. For cardiac triglyceride assay, 10–20 mg heart tissue was minced into small pieces and then homogenized in standard diluent assay reagent (10 μl/mg tissue). Tissue homogenate was centrifuged at 10,000 × g for 10 min. at 4°C, and 10 μl supernatant was used for triglyceride assay.

### Histopathological examination

Paraffin sections (5 μm) from cardiac tissue dissected from mice were stained with haematoxylin and eosin and observed under light microscopy as described before 26.

### Oil Red O staining

Lipid accumulation was evaluated by Oil Red O staining as described previously 27. Cryosections (10 μm thick) from heart tissue embedded in optimal cutting temperature medium (Tissue-Tek® O.C.T™ Compound, Sakura, Torrance, CA, USA) were fixed in 10% buffered formalin for 30 min. at room temperature and stained with Oil Red O for 1 hr. After washing with 60% isopropanol, the sections were then counterstained with haematoxylin (DAKO, Carpinteria, CA, USA) for 30 sec. A Nikon Eclipse E600 microscope (Nikon, Melville, NY, USA) was used to capture the Oil Red O-stained tissue sections at 40× magnification.

### Sirius Red staining

Sirius Red staining for collagen deposition was used to determine cardiac fibrosis as described previously 25. Briefly, 5 μm paraffin-embedded heart tissue sections were stained with 0.1% Sirius Red F3BA and 0.25% Fast Green FCF. The proportion of collagen in Sirius Red-stained sections was then evaluated using a Nikon Eclipse E600 microscopy system.

### Real-time quantitative polymerase chain reaction (RT-qPCR)

Total RNA was extracted from heart tissue using TRIzol reagent (Invitrogen, Carlsbad, CA, USA). After quantified using a Nanodrop ND-1000 spectrophotometer, 1 μg total RNA was used to synthesize first-strand complimentary DNA (cDNA) using reverse transcription kit (Promega, WI, USA) as described before 28. RT-qPCR was carried out with the ABI 7300 real-time PCR system (Applied Biosystems, Grand Island, NY, USA) in a 20 μl reaction system containing 10 μl of TaqMan Universal PCR Master Mix, 1 μl of primers, 6 μl ddH_2_O and 3 μl of cDNA (1:4 dilution with nuclease-free water). Primers for mouse glyceraldehyde 3-phosphate dehydrogenase (GAPDH) and FGFR1 (Invitrogen) were used for RT-qPCR assay.

### Western blot

Total proteins from heart tissue were fractionated on 10% SDS-PAGE gels and transferred to a nitrocellulose membrane. The membrane was blocked with 5% non-fat milk for 1 hr, and incubated overnight at 4°C on a rocking platform with the following primary antibodies: anti-phosphor-AMP-activated protein kinase (AMPK)α (Thr172), anti-AMPKα (Cell Signaling, Danvers, MA, USA), anti-CD36, anti-peroxisome proliferator- activated receptor gamma co-activator 1alpha (PGC1α; Abcam, MA, USA), anti-connective tissue growth factor (CTGF), anti-hexokinase II (HKII), anti-nuclear factor (erythroid-derived 2)-like 2 (Nrf2) and anti-GAPDH (Santa Cruz Biotechnology, Dallas, TX, USA). After unbound antibodies were washed out with tris-buffered saline (pH 7.2) containing 0.05% Tween20, membranes were incubated with corresponding secondary antibody for 1 hr at room temperature. Antigen-antibody complexes were visualized with an enhanced chemiluminescence detection kit (Thermo Scientific, Waltham, MA, USA). Quantitative densitometry was performed on the identified bands by using a computer-based measurement system as performed in previous studies 25,29.

### Statistical analysis

Data were collected from five or more mice per group and presented as mean ± SD. One-way anova was used to determine general difference, followed by a *post hoc* Turkey’s test for the difference between groups, using Origin 7.5 laboratory data analysis and graphing software. Statistical significance was considered as *P* < 0.05.

## Results

### FGF21 expression decreased under type 1 diabetes conditions

**Figure 1:**
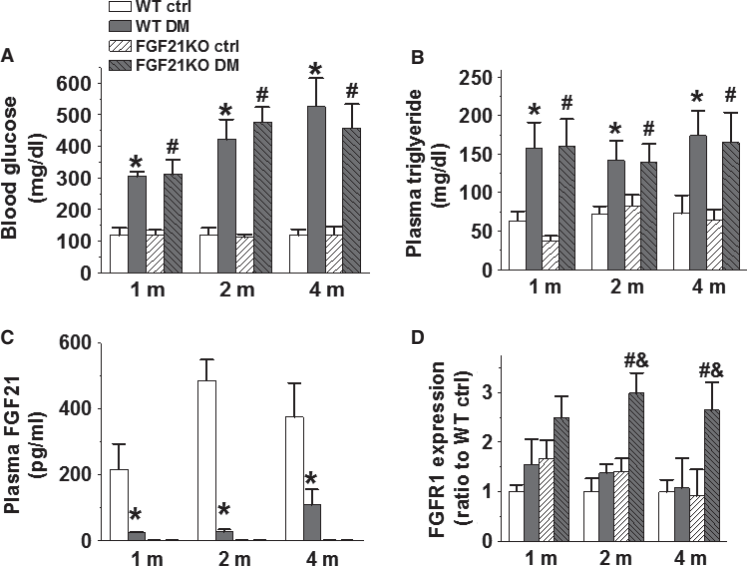
Diabetes-induced high blood glucose and triglyceride levels, and FGF21 deficiency. At indicated time-points after diabetes onset, fasting blood glucose was detected using a SureStep complete blood glucose monitor (A), plasma triglyceride level was measured using a triglyceride assay kit (B), and plasma FGF21 was assayed using a FGF21 Quantikine Elisa kit (C). The expression of cardiac FGFR1 mRNA was detected by RT-qPCR and GAPDH was used as the loading control. Three duplications were set for each sample (D). Data are presented as means ± SD (*n* ≥ 5 for each group). *P < 0.05 *versus* WT Ctrl group; ^#^*P* < 0.05 *versus* FGF21KO Ctrl group; ^&^*P* < 0.05 *versus* WT DM group. Ctrl: control; DM: diabetes mellitus; WT: wild-type; FGF21KO: FGF21 knockout; m: month(s). [Correction added on 12 May 2015 after first online publication: Panel D was inserted in Figure 1]

Wild-type and FGF21KO diabetic mice showed similar, persistent increases in whole blood glucose and plasma triglyceride levels up until organ harvested 4 months after STZ-induced diabetes onset (Fig.1A and B). To elucidate the relationship between FGF21 and DCM, we measured the plasma levels of FGF21 and the cardiac mRNA levels of its preferred receptor FGFR1 in both WT and FGF21KO mice under diabetic and non-diabetic conditions. Plasma FGF21 level in WT diabetic mice significantly decreased at 1, 2 and 4 months after diabetes onset (Fig.1C), which was accompanied by obvious trend of cardiac FGFR1 mRNA level up-regulation at 1 and 2 months after DM onset (Fig.1D), indicating that type 1 diabetes systematically down-regulation of FGF21 resulted in its receptor compensative up-regulation in cardiac tissue. No obvious FGF21 expression was observed under diabetic and non-diabetic conditions in FGF21KO mice, as expected (Fig.1C). The deletion of FGF21 was accompanied by a slight compensative up-regulation of cardiac FGFR1 mRNA under basal conditions, which was significantly amplified by diabetes in FGF21KO mice (Fig.1D).

### FGF21 deletion-aggravated diabetes-induced cardiac dysfunction

**Figure 2:**
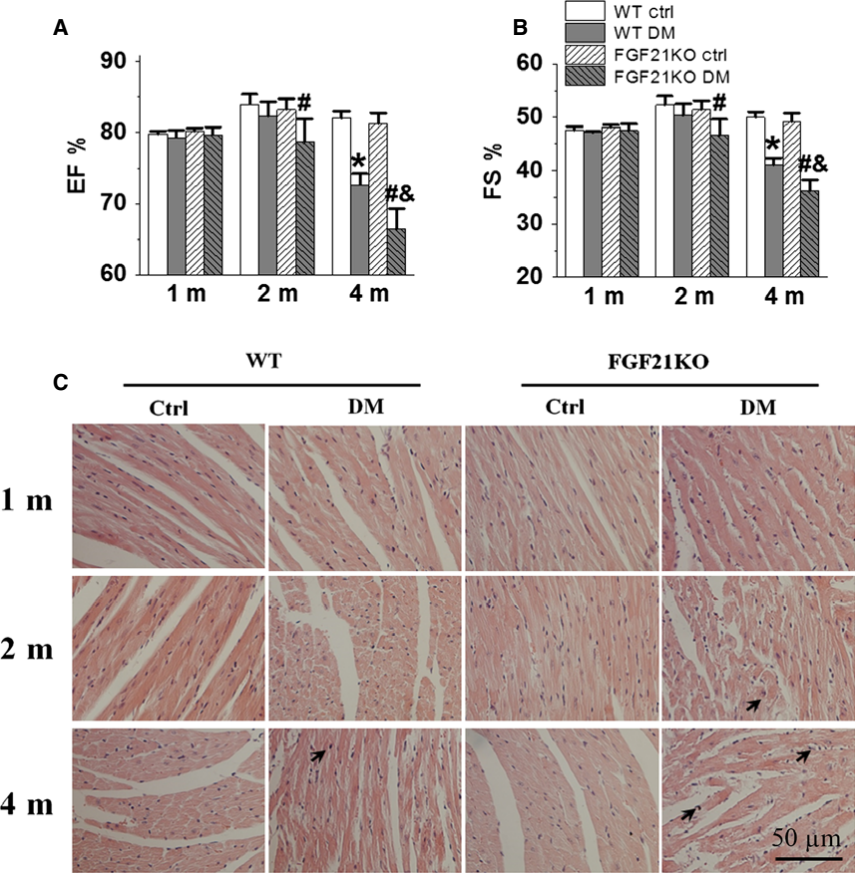
FGF21 deletion-aggravated diabetes-induced cardiac dysfunction. At indicated time-points after diabetes onset, cardiac function was evaluated by transthoracic echocardiography and expressed as LV ejection fraction (EF%, A) and fraction shortening (FS%, B). Myocardial structural damage indicated by haematoxylin and eosin staining (C). Paraffin-embedded heart tissue was stained with haematoxylin and eosin and examined under light microscope. Arrows indicate cardiac cell death with karyorrhexis, pyknosis and/or karyolysis. Magnification = 400 × . Data are presented as means ± SD (*n* ≥ 5 for each group). *P < 0.05 *versus* WT Ctrl group; ^#^*P* < 0.05 *versus* FGF21KO Ctrl group; ^&^*P* < 0.05 *versus* WT DM group. The abbreviations are same as in Figure1.

The role of FGF21 in the development of DCM was investigated by determining the effect of FGF21 deletion on cardiac structure and function in diabetic mice. Under basal conditions, FGF21KO mice did not show marked alterations in heart weight (Fig. S1), cardiac structure and function (Fig.2 and Table S1). However, both WT and FGF21KO diabetic mice showed heart weight decrease at 1, 2, and 4 months after diabetes onset (Fig. S1). WT diabetic mice did not exhibit cardiac dysfunction until 4 months after diabetes, reflected by decreased ejection fraction and fraction shortening (Fig.2). FGF21KO diabetic mice developed cardiac dysfunction at 2 months after diabetes onset; at 4 months, FGF21KO diabetic mice showed more severe cardiac functional impairment than WT diabetic mice (Fig.2). Parameters of cardiac structure showed similar patterns of change (Table S1). At 4 months, WT diabetic mice showed decrease in diastolic and systolic LVPW and systolic LVID, and an increase in systolic LV volume compared to WT control mice, and FGF21KO diabetic mice showed decrease in systolic and diastolic IVS and LVPW, and an increase in diastolic LVID and systolic LVID. Moreover, FGF21KO diabetic mice had dramatically lower systolic IVS and diastolic and systolic LVPW, and higher systolic LVID and diastolic and systolic LV volume than WT diabetic mice (Table S1). These results demonstrate that the cardiac dysfunction developed in FGF21KO diabetic mice was earlier and more severe than that in WT diabetic mice, indicating that deletion of FGF21 aggravated the development of DCM.

Histological examination revealed severe pathological changes in the hearts of FGF21KO diabetic mice in comparison with that of FGF21KO control and WT diabetic mice. As shown in Figure2C, both FGF21KO and WT control hearts had regular and intact myocardial arrangements and clearly visible nuclei, and WT diabetic hearts showed certain irregularity of the myocardial fibres, especially at 4 months after diabetes onset, while FGF21KO diabetic hearts exhibited large areas of irregular myocardial arrangements, myofibrillar discontinuation and cell death (karyorrhexis, pyknosis and/or karyolysis).

Blood pressure was detected using a CODA™ mouse tail-cuff system, and no significant changes in systolic and diastolic pressure were observed in both WT and FGF21KO mice under diabetic and non-diabetic conditions (Fig. S2), which indicated that FGF21 deletion did not affect blood pressure under basal and experimental diabetic conditions. The above-mentioned diabetes-induced cardiac dysfunction was independent of blood pressure changes.

### FGF21 deletion accelerated diabetes-induced cardiac remodelling

**Figure 3:**
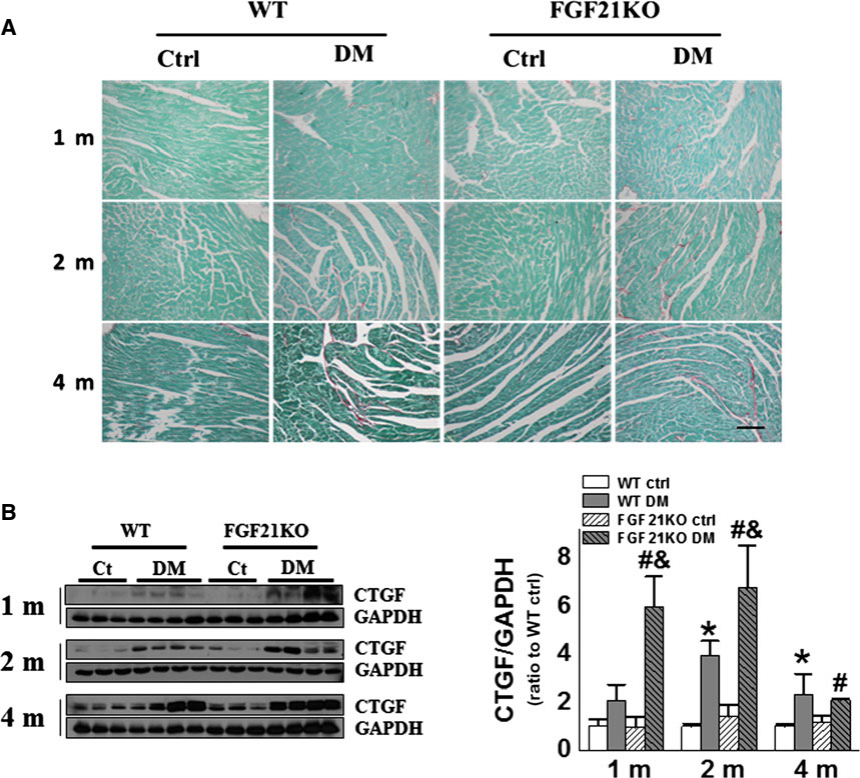
FGF21 deletion accelerated diabetes-induced cardiac remodelling. At indicated time-points after diabetes onset, cardiac fibrosis was evaluated by Sirius Red staining of collagen accumulation (A) and Western blot of CTGF expression (B). Data are presented as means ± SD (*n* ≥ 5 for each group). *P < 0.05 *versus* WT Ctrl group; ^#^*P* < 0.05 *versus* FGF21KO Ctrl group; ^&^*P* < 0.05 *versus* WT DM group; bar = 100 μm. The abbreviations are same as in Figure1.

FGF21KO mice did not show marked cardiac fibrosis compared to WT mice under normal conditions, but diabetes-induced significant cardiac remodelling. Collagen accumulation (Fig.3A) and expression of fibrotic mediator CTGF (Fig.3B) were significantly increased in the heart of WT diabetic mice from 2 months, and FGF21KO diabetic mice from 1 month after diabetes onset. The elevation of collagen accumulation and CTGF expression was higher in FGF21KO diabetic mice than that in WT diabetic mice at 2 months after diabetes (Fig.3A and B), indicating that FGF21 deletion accelerated and aggravated diabetes-induced cardiac fibrotic remodelling.

### FGF21 deletion-exacerbated diabetes-induced cardiac oxidative stress

**Figure 4:**
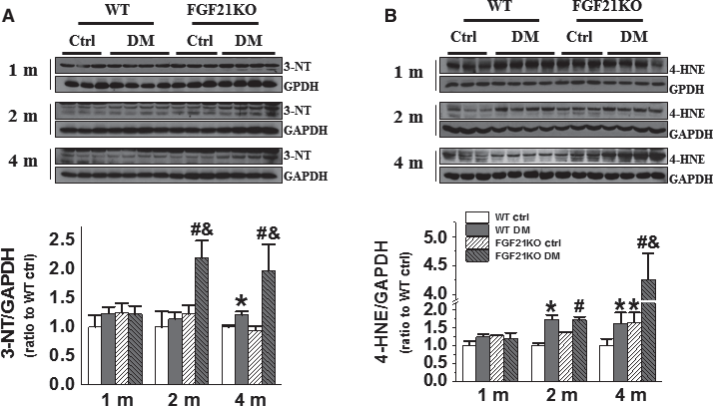
FGF21 deletion-exacerbated diabetes-induced cardiac oxidative stress. At indicated time-points after diabetes onset, the markers of cardiac oxidative stress including 3-NT (A) and 4-HNE (B) were evaluated by Western blot. Data are presented as means ± SD (*n* ≥ 5 for each group). *P < 0.05 *versus* WT Ctrl group; ^#^*P* < 0.05 *versus* FGF21KO Ctrl group; ^&^*P* < 0.05 *versus* WT DM group. The abbreviations are same as in Figure1.

Diabetic hyperglycaemia- and hyperlipidaemia-induced oxidative stress plays a critical role in the development of DCM 29. Consequently, cardiac oxidative status was evaluated by the expression of 3-NT and 4-HNE as in our previous report 30. The expression of 3-NT remained unchanged in FGF21KO mice under basal conditions, but was elevated under diabetic conditions (Fig.4A). It was elevated only at 4 months in WT diabetic mice, but at both 2 and 4 months after diabetes onset in FGF21KO diabetic mice (Fig.4A). Moreover, the 3-NT expression in FGF21KO diabetic mice was significantly higher than that of WT diabetic mice at 2 and 4 months after diabetes onset (Fig.4A). The expression of 4-HNE showed similar change pattern with that of 3-NT, both FGF21KO and WT diabetic mice exhibited higher 4-HNE expression than their controls at 2 and 4 months after diabetes onset, but FGF21KO diabetic mice had higher 4-HNE expression than WT diabetic mice at 4 months after diabetes onset (Fig.4B).

### FGF21 deletion-aggravated diabetes-induced cardiac lipid accumulation

**Figure 5:**
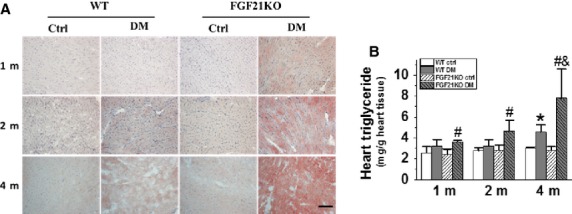
FGF21 deletion-aggravated diabetes-induced cardiac lipid accumulation. At indicated time-points after diabetes onset, cardiac lipid accumulation was evaluated by Oil Red O staining (A) and triglyceride content (B). Data are presented as means ± SD (*n* ≥ 5 for each group). *P < 0.05 *versus* WT Ctrl group; ^#^*P* < 0.05 *versus* FGF21KO Ctrl group; ^&^*P* < 0.05 *versus* WT DM group; bar = 100 μm. The abbreviations are same as in Figure1.

Fibroblast growth factor 21 was identified as a potent metabolic regulator for lipid metabolism in several organs 6. We therefore quantified cardiac lipid accumulation by Oil Red O staining (Fig.5A) and triglyceride assay (Fig.5B). No obvious cardiac lipid accumulation was observed in both WT and FGF21KO mice under basal conditions (Fig.5A and B). Diabetes-induced significant cardiac lipid accumulation in WT mice only at 4 months, but in the FGF21KO mice starting from 1 month until 4 months after diabetes onset (Fig.5A and B). Moreover, FGF21KO diabetic mice exhibited more severe cardiac lipid accumulation at 2 and 4 months after diabetes onset than WT diabetic mice (Fig.5A and B). These results demonstrate that FGF21 deletion accelerated and exacerbated cardiac lipid accumulation under diabetic conditions.

### FGF21 deletion accelerated diabetes-induced cardiac CD36 and Nrf2 up-regulation and PGC1α down-regulation

**Figure 6:**
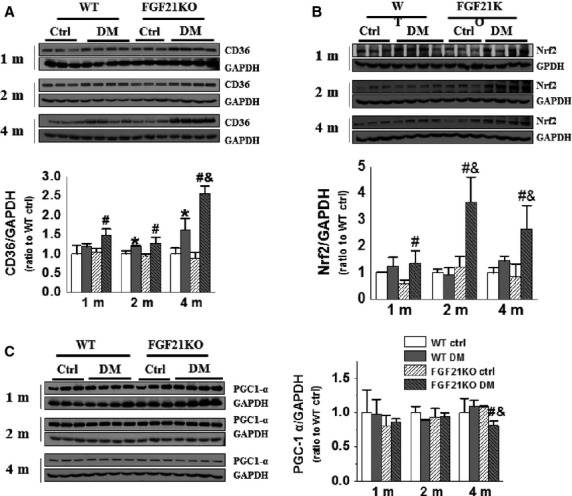
FGF21 deletion accelerated diabetes-induced cardiac CD36 and Nrf2 up-regulation and PGC1α down-regulation. At indicated time-points after diabetes onset, the expression of CD36 (A), Nrf2 (B) and PGC1α (C) were detected by Western blot. Data are presented as means ± SD (*n* ≥ 5 for each group). *P < 0.05 *versus* WT Ctrl group; ^#^*P* < 0.05 *versus* FGF21KO Ctrl group; ^&^*P* < 0.05 *versus* WT DM group. The abbreviations are same as in Figure1.

To uncover how FGF21 deletion affects cardiac lipid accumulation, the expression of CD36, a critical regulator of fatty acid transport 31, was detected by Western blot. FGF21 deletion had no obvious effects on cardiac CD36 expression under basal conditions, but diabetes significantly up-regulated CD36 expression in both WT and FGF21KO hearts (Fig.6A). Cardiac CD36 expression was elevated from 2 months in WT diabetic mice and from 1 month in FGF21KO diabetic mice after diabetes onset. At 4 months after diabetes onset, cardiac CD36 expression in FGF21KO diabetic mice was significantly higher than that of WT diabetic mice (Fig.6A). Reportedly, Nrf2-mediated CD36 up-regulation plays critical role in lipid metabolism in macrophage, aorta and liver tissues 32–34. So, we detected cardiac Nrf2 expression by Western blot (Fig.6B). We found that cardiac Nrf2 expression showed no difference between FGF21KO mice and WT mice under basal conditions (Fig.6B). Under diabetic conditions, Nrf2 expression was slightly, but not significantly, elevated in WT mice; but progressively increased from 1 to 4 months after diabetes onset in FGF21KO mice (Fig.6B). These results indicate that FGF21 deletion-exacerbated diabetes-induced cardiac Nrf2 and CD36 up-regulation.

PGC1α, an essential regulator of fatty acid oxidation 35, was also detected by Western blot. The cardiac PGC1α expression was not affected by FGF21 deletion under basal conditions, but was significantly attenuated by diabetes in FGF21KO diabetic mice at 4 months after diabetes onset (Fig.6C). No significant changes of cardiac PGC1α expression were observed under either diabetic or non-diabetic conditions in WT mice (Fig.6C). The elevation of Nrf2 and CD36, and decline of PGC1α expression imply that FGF21 deletion increased lipid uptake and decreased lipid oxidation leading to sub-optimal cardiac lipid metabolism under diabetic conditions, which resulted in aggravated cardiac lipid accumulation in FGF21KO diabetic mice.

### FGF21 deletion accelerated diabetes-induced cardiac glucose metabolism impairment

**Figure 7:**
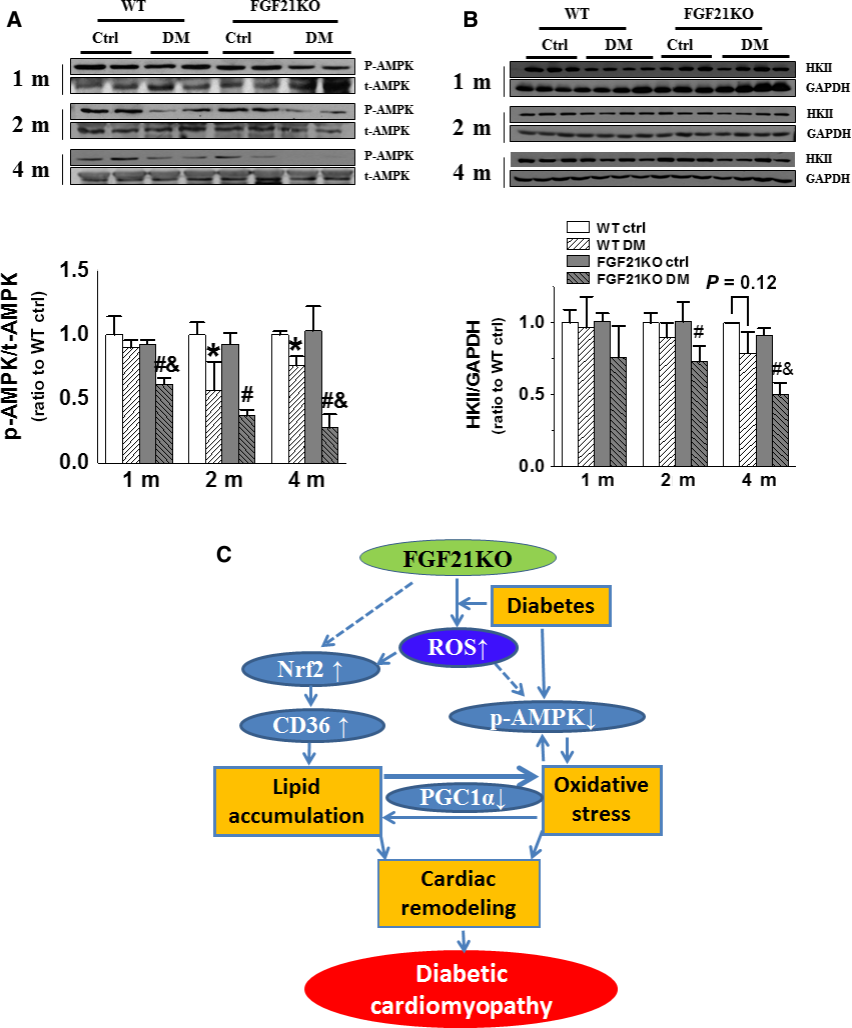
FGF21 deletion accelerated diabetes-induced cardiac glucose metabolism impairment. At indicated time-points after diabetes onset, cardiac AMPK phosphorylation (A) and HKII expression (B) were detected by Western blot. Data are presented as means ± SD (*n* ≥ 5 for each group). *P < 0.05 *versus* WT Ctrl group; ^#^*P* < 0.05 *versus* FGF21KO Ctrl group; ^&^*P* < 0.05 *versus* WTDM group. The proposed mechanism of FGF21 deletion leading to aggravated DCM is schematically described (C): FGF21 deletion aggravates the ROS accumulation induced by diabetes, which in turn increases Nrf2 expression. Nrf2 up-regulation elevates CD36 expression, and induces fatty acid over-uptake and cardiac lipid accumulation. In addition, FGF21 deletion-mediated diabetic ROS accumulation attenuates the phosphorylation of AMPK, which leads to impairment of cardiac glucose metabolism and cardiac energy metabolic balance and further aggravates cardiac oxidative stress. The elevated cardiac lipid accumulation and oxidative stress synergistically down-regulate PGC1α function and induce cardiac remodelling, and eventually accelerate the development of DCM.

In addition to lipid, glucose metabolism is another important source of energy for heart contraction. Reportedly, FGF-21 is also a potent glucose metabolic regulator in several organs including the heart 6, and AMPK is a sensor of energy homoeostasis and a regulator of glucose uptake and fatty acid β-oxidation 36. So, glucose metabolism in cardiac tissue was also investigated to evaluate the effect of FGF21 deletion. FGF21KO mice did not show marked alterations in phosphorylation of AMPK under basal conditions (Fig.7A). Significant down-regulation of cardiac AMPK phosphorylation was observed from 2 months in WT diabetic mice, and from 1 month after diabetes onset in FGF21KO diabetic mice. Moreover, the cardiac AMPK phosphorylation in FGF21KO diabetic mice was significantly lower than that of WT diabetic mice at 1 and 4 months after diabetes onset (Fig.7A). The expression of HKII, an enzyme that catalyses the first step of glycolysis by conversion of glucose to glucose-6-phosphate, was also detected. The cardiac HKII expression was not affected by FGF21 deletion under basal conditions, but was significantly impaired by diabetes in FGF21KO diabetic mice at 2 and 4 months after diabetes onset (Fig.7B). No significant changes of cardiac HKII expression were observed under both diabetic and non-diabetic conditions in WT mice (Fig.7B). These results indicate that FGF21 deletion-aggravated diabetes-induced impairment of cardiac glucose metabolism and cardiac energy metabolic balance in FGF21KO diabetic mice.

## Discussion

Fibroblast growth factor 21 is a newly discovered metabolic hormone. In addition to its essential roles in regulating glucose and lipid metabolism through pleiotropic actions in liver and adipocyte tissues, FGF21 also plays critical role in cardiac pathogenesis. In this study, for the first time we established that FGF21 deletion is susceptible to develop DCM in STZ-induced type 1 diabetic mice, which is predominantly attributed to the exacerbated cardiac lipid accumulation *via* Nrf2 up-regulation of CD36-mediated cardiac fatty acid accumulation.

In STZ- induced type 1 diabetes in this study, circulating FGF21 reduced from 1 month to the experimental termination at 4 months after diabetes onset (Fig.1C), which was consistent with a previous report that serum FGF21 levels were significantly lower in type 1 diabetic patients than that of control subjects 37. It was also found that decreased FGF21 levels were accompanied by significant cardiac dysfunction, remodelling and oxidative stress at 4 months after diabetes onset in WT diabetic mice, while FGF21 complete deletion significantly accelerated and aggravated the above-mentioned cardiac structural, functional and oxidative stress changes in FGF21KO diabetic mice (Figs4 and Table S1), which indicate that FGF21 plays a critical role in protecting the heart against the development of DCM under experimental type 1 diabetic conditions.

Since diabetic down-regulation of plasma FGF21 levels (Fig.1C) was accompanied by an obvious trend of compensative up-regulation of cardiac FGFR1 mRNA levels in WT mice (Fig.1D), while the deletion of FGF21 was also accompanied by a slight compensative up-regulation of cardiac FGFR1 mRNA levels under basal conditions, which was significantly amplified under diabetic condition in FGF21KO mice (Fig.1D). These results imply that the exacerbation of DCM by the *fgf21* gene deletion might directly attribute to the dysfunction of FGF21/FGFR1 axis.

Fibroblast growth factor 21 was reported to have anti-hyperglycemic and anti-hyperlipidemic properties in diabetic rodent 13 and monkey 14 models, and hyperglycaemia and hyperlipidaemia were thought to be the major contributors to DCM 2. Thus, we assumed that FGF21 deletion might further elevate plasma glucose and triglyceride levels, contributing to the accelerated and aggravated development of DCM in FGF21KO diabetic mice. Unexpectedly, both FGF21KO and WT diabetic mice have typically diabetic hyperglycaemia and hyperlipidaemia, no significant differences were observed in plasma glucose (Fig.1A) and triglyceride (Fig.1B) levels between FGF21KO and WT diabetic mice. This implies that the dramatic diabetes-induced down-regulation of plasma FGF21 levels in WT type 1 diabetic mice (Fig.1C) were comparable to FGF21 deletion in FGF21KO diabetic mice with respect to the whole body glucose and lipid metabolic regulation by FGF21. The elevated serum FGF21 was reported to associate with hypertension 38. But in the present study, FGF21KO mice did not exhibit alteration in blood pressure under either basal or diabetic conditions (Fig. S2).

Cardiac lipid accumulation plays a causative role in the development of DCM 39. FGF21 was also reported to regulate lipid homoeostasis in liver 40, adipose tissue 41 and kidney 27. In the present study, we observed that FGF21 deletion significantly aggravated cardiac lipid accumulation (Fig.5), which was time-dependently associated with diabetes-accelerated cardiac dysfunction (Fig.2) and remodelling (Fig.3) in FGF21KO diabetic mice, indicating that FGF21 plays a critical role in cardiac protection from the development of DCM by regulation of cardiac lipid metabolism under type 1 diabetic conditions.

CD36 is a pivotal lipid transport protein that mediates fatty acid transport and utilization in the heart 42. CD36 is believed to play a critical role in intramyocardial lipid accumulation, fatty acid and glucose oxidation and in the subsequent deterioration in cardiac ATP supply in age-induced cardiomyopathy in mice 43. Under physiological conditions, increased cardiac CD36 expression can compensate for the decreased supply of long-chain fatty acid 44; but under diabetic conditions, the elevated cardiac CD36 expression mediates excess uptake of fatty acid leading to cardiac lipid accumulation 31. In the present study, cardiac CD36 expression was further up-regulated in FGF21KO diabetic mice (Fig.6A), which was time-dependently associated with excess cardiac lipid accumulation (Fig.5A and B). Meanwhile, cardiac AMPK phosphorylation (Fig.7A), an indicator of energy homoeostasis, and HKII expression (Fig.7B), an indicator of glucose utilization, were also time-dependently further decreased in FGF21KO diabetic mice. Moreover, PGC1α, a critical regulator of fatty acid β-oxidation and a key mediator of FGF21 regulation of lipid metabolism 45 was significantly decreased only in the heart of FGF21KO diabetic mice at the late stage of DCM (Fig.6C). These results imply that FGF21 deletion-induced excess cardiac lipid uptake and lipid accumulation might impair cardiac lipid and glucose utilization and energetic balance, which further exacerbated lipid accumulation and impaired lipid β-oxidation, contributing to the accelerated DCM in FGF21KO diabetic mice.

CD36 expression was strictly regulated in the heart tissue 46. Accumulating evidence indicate that Nrf2 up-regulation of CD36-mediated lipid uptake and excess accumulation in macrophages and smooth muscle cells play critical roles in the development of atherosclerosis 32–34. Consistent with these previous studies, cardiac Nrf2 expression was also found to be largely elevated in FGF21KO diabetic mice, but not in WT diabetic mice (Fig.6B), which were time-dependently associated with the up-regulation of cardiac CD36 expression (Fig.6A) and cardiac lipid accumulation (Fig.5A and B) in the present study. Even though there was one report that has identified Nrf2 as a novel regulator repressing FGF21 expression in liver and adipose tissue under long-term high-fat diet-induced obese conditions 47, further study to dissect the mechanism of FGF21 deletion-mediated up-regulation of cardiac Nrf2-driven CD36 expression and lipid accumulation under type 1 diabetic conditions is warranted.

Among diabetic patients, 90–95% suffer from type 2 diabetes 48,49. A limitation of the present study is the lack of the relevance to type 2 diabetes. It has been generally accepted that DCM has similar pathological mechanisms in both type 1 and type 2 diabetes 50, but FGF21 has been demonstrated to have different changing patterns of serum level increase in type 2 diabetes and decrease in type 1 diabetes 37. To ensure a greater clinical relevance, whether FGF21 plays a similar role in the development of DCM in type 1 and type 2 diabetes really needs to be comparatively studied in the future.

In conclusion, FGF21 deletion up-regulation of Nrf2-driven CD36 expression exacerbates cardiac lipid uptake and accumulation, which in turn impairs cardiac lipid and glucose utilization and cardiac energy balance, and aggravates cardiac oxidative stress, eventually accelerating the development of DCM (Fig.7C). FGF21 deletion-aggravated DCM indicates that FGF21 may be a therapeutic target for the treatment of diabetic cardiovascular complications.

## Supporting Information

**Fig. S1.** Heart weight **Fig. S2.** Blood pressure **Table S1**. Heart function

## References

[b1] Rubler S, Yuceoglu YZ, Kumral T (1972). New type of cardiomyopathy associated with diabetic glomerulosclerosis. Am J Cardiol.

[b2] Boudina S, Abel ED (2007). Diabetic cardiomyopathy revisited. Circulation.

[b3] Taegtmeyer H, McNulty P, Young ME (2002). Adaptation and maladaptation of the heart in diabetes: part I: general concepts. Circulation.

[b4] McGavock JM, Victor RG, Unger RH (2006). Adiposity of the heart, revisited. Ann Intern Med.

[b5] Nishimura T, Nakatake Y, Konishi M (2000). Identification of a novel FGF, FGF-21, preferentially expressed in the liver. Bba-Gene Struct Expr.

[b6] Adams AC, Kharitonenkov A (2012). FGF21: the center of a transcriptional nexus in metabolic regulation. Curr Diabetes Rev.

[b7] Tacer KF, Bookout AL, Ding XS (2010). Research resource: comprehensive expression atlas of the fibroblast growth factor system in adult mouse. Mol Endocrinol.

[b8] Muise ES, Azzolina B, Kuo DW (2008). Adipose fibroblast growth factor 21 is up-regulated by peroxisome proliferator-activated receptor gamma and altered metabolic states. Mol Pharmacol.

[b9] Hondares E, Iglesias R, Giralt A (2011). Thermogenic activation induces FGF21 expression and release in brown adipose tissue. J Biol Chem.

[b10] Kim KH, Jeong YT, Oh H (2013). Autophagy deficiency leads to protection from obesity and insulin resistance by inducing Fgf21 as a mitokine. Nat Med.

[b11] Iglesias P, Selgas R, Romero S (2012). Biological role, clinical significance, and therapeutic possibilities of the recently discovered metabolic hormone fibroblastic growth factor 21. Eur J Endocrinol.

[b12] Fisher FM, Kleiner S, Douris N (2012). FGF21 regulates PGC-1 alpha and browning of white adipose tissues in adaptive thermogenesis. Gene Dev.

[b13] Kharitonenkov A, Shiyanova TL, Koester A (2005). FGF-21 as a novel metabolic regulator. J Clin Invest.

[b14] Kharitonenkov A, Wroblewski VJ, Koester A (2007). The metabolic state of diabetic monkeys is regulated by fibroblast growth factor-21. Endocrinology.

[b15] Hotta Y, Nakamura H, Konishi M (2009). Fibroblast growth factor 21 regulates lipolysis in white adipose tissue but is not required for ketogenesis and triglyceride clearance in liver. Endocrinology.

[b16] Lin Z, Tian H, Lam KSL (2013). Adiponectin mediates the metabolic effects of FGF21 on glucose homeostasis and insulin sensitivity in mice. Cell Metab.

[b17] Zhao Y, Dunbar JD, Kharitonenkov A (2012). FGF21 as a therapeutic reagent. Adv Clin Exp Med.

[b18] Sarruf DA, Thaler JP, Morton GJ (2010). Fibroblast growth factor 21 action in the brain increases energy expenditure and insulin sensitivity in obese rats. Diabetes.

[b19] Berglund ED, Li CY, Bina HA (2009). Fibroblast growth factor 21 controls glycemia *via* regulation of hepatic glucose flux and insulin sensitivity. Endocrinology.

[b20] Mraz M, Bartlova M, Lacinova Z (2009). Serum concentrations and tissue expression of a novel endocrine regulator fibroblast growth factor-21 in patients with type 2 diabetes and obesity. Clin Endocrinol.

[b21] Suzuki M, Uehara Y, Motomura-Matsuzaka K (2008). beta Klotho is required for fibroblast growth factor (FGF) 21 signaling through FGF receptor (FGFR) 1c and FGFR3c. Mol Endocrinol.

[b22] Kurosu H, Choi M, Ogawa Y (2007). Tissue-specific expression of beta Klotho and fibroblast growth factor (FGF) receptor Isoforms determines metabolic activity of FGF19 and FGF21. J Biol Chem.

[b23] Liu SQ, Roberts D, Kharitonenkov A (2013). Endocrine protection of ischemic myocardium by FGF21 from the liver and adipose tissue. Sci Rep.

[b24] Planavila A, Redondo I, Hondares E (2013). Fibroblast growth factor 21 protects against cardiac hypertrophy in mice. Nat Commun.

[b25] Zhou G, Li X, Hein DW (2008). Metallothionein suppresses angiotensin II-induced nicotinamide adenine dinucleotide phosphate oxidase activation, nitrosative stress, apoptosis, and pathological remodeling in the diabetic heart. J Am Coll Cardiol.

[b26] Tan Y, Li Y, Xiao J (2009). A novel CXCR4 antagonist derived from human SDF-1beta enhances angiogenesis in ischaemic mice. Cardiovasc Res.

[b27] Zhang C, Shao ML, Yang H (2013). Attenuation of hyperlipidemia- and diabetes-induced early-stage apoptosis and late-stage renal dysfunction *via* administration of fibroblast growth factor-21 is associated with suppression of renal inflammation. PLoS ONE.

[b28] Miao X, Wang YG, Sun J (2013). Zinc protects against diabetes-induced pathogenic changes in the aorta: roles of metallothionein and nuclear factor (erythroid-derived 2)-like 2. Cardiovasc Diabetol.

[b29] Tan Y, Ichikawa T, Li J (2011). Diabetic downregulation of Nrf2 activity *via* ERK contributes to oxidative stress-induced insulin resistance in cardiac cells *in vitro* and *in vivo*. Diabetes.

[b30] Bai Y, Cui W, Xin Y (2013). Prevention by sulforaphane of diabetic cardiomyopathy is associated with up-regulation of Nrf2 expression and transcription activation. J Mol Cell Cardiol.

[b31] Greenwalt DE, Scheck SH, Rhinehartjones T (1995). Heart Cd36 expression is increased in murine models of diabetes and in mice fed a high-fat diet. J Clin Invest.

[b32] Bozaykut P, Karademir B, Yazgan B (2014). Effects of vitamin E on peroxisome proliferator-activated receptor gamma and nuclear factor-erythroid 2-related factor 2 in hypercholesterolemia-induced atherosclerosis. Free Radical Biol Med.

[b33] Ishii T, Itoh K, Ruiz E (2004). Role of Nrf2 in the regulation of CD36 and stress protein expression in murine macrophages - Activation by oxidatively modified LDL and 4-hydroxynonenal. Circ Res.

[b34] More VR, Xu JL, Shimpi PC (2013). Keap1 knockdown increases markers of metabolic syndrome after long-term high fat diet feeding. Free Radical Bio Med.

[b35] Wang Y, Feng W, Xue W (2009). Inactivation of GSK-3beta by metallothionein prevents diabetes-related changes in cardiac energy metabolism, inflammation, nitrosative damage, and remodeling. Diabetes.

[b36] Hardie DG, Ross FA, Hawley SA (2012). AMPK: a nutrient and energy sensor that maintains energy homeostasis. Nat Rev Mol Cell Bio.

[b37] Xiao Y, Xu AM, Law LSC (2012). Distinct changes in serum fibroblast growth factor 21 levels in different subtypes of diabetes. J Clin Endocrinol Metab.

[b38] Semba RD, Crasto C, Strait J (2013). Elevated serum fibroblast growth factor 21 is associated with hypertension in community-dwelling adults. J Hum Hypertens.

[b39] Goldberg IJ, Trent CM, Schulze PC (2012). Lipid metabolism and toxicity in the heart. Cell Metab.

[b40] Badman MK, Pissios P, Kennedy AR (2007). Hepatic fibroblast growth factor 21 is regulated by PPAR alpha and is a key mediator of hepatic lipid metabolism in ketotic states. Cell Metab.

[b41] Muise ES, Souza S, Chi A (2013). Downstream signaling pathways in mouse adipose tissues following acute *in vivo* administration of fibroblast growth factor 21. PLoS ONE.

[b42] Koonen DPY, Glatz JFC, Bonen A (2005). Long-chain fatty acid uptake and FAT/CD36 translocation in heart and skeletal muscle. Bba-Mol Cell Biol L.

[b43] Koonen DPY, Febbraio M, Bonnet S (2007). CD36 expression contributes to age-induced cardiomyopathy in mice. Circulation.

[b44] Luiken JJFP, Coort SLM, Koonen DPY (2004). Regulation of cardiac long-chain fatty acid and glucose uptake by translocation of substrate transporters. Pflug Arch Eur J Phy.

[b45] Potthoff MJ, Inagaki T, Satapati S (2009). FGF21 induces PGC-1 alpha and regulates carbohydrate and fatty acid metabolism during the adaptive starvation response. Proc Natl Acad Sci USA.

[b46] Chen M, Yang YK, Loux TJ (2006). The role of hyperglycemia in FAT/CD36 expression and function. Pediatr Surg Int.

[b47] Chartoumpekis DV, Ziros PG, Psyrogiannis AI (2011). Nrf2 represses FGF21 during long-term high-fat diet-induced obesity in mice. Diabetes.

[b48] Georgescu A (2011). Vascular dysfunction in diabetes: the endothelial progenitor cells as new therapeutic strategy. World J Diabetes.

[b49] Yan J, Tie G, Park B (2009). Recovery from hind limb ischemia is less effective in type 2 than in type 1 diabetic mice: roles of endothelial nitric oxide synthase and endothelial progenitor cells. J Vasc Surg.

[b50] Bugger H, Abel ED (2009). Rodent models of diabetic cardiomyopathy. Dis Mod Mech.

