# Supplementary material for: FGF21 deletion exacerbates diabetic cardiomyopathy by aggravating cardiac lipid accumulation
Source: J Cell Mol Med. 2015 Mar 30;19(7):1557–68. doi: 10.1111/jcmm.12530 (PMC4511354; doi:10.1111/jcmm.12530)
Supplement: Supplementary file 1 [file jcmm0019-1557-sd1.docx]

**FGF21 deletion exacerbates diabetic cardiomyopathy by aggravating cardiac lipid accumulation**

Xiaoqing Yan^1, 2^, Jun Chen^1, 2, 3^, Chi Zhang^1^, Shanshan Zhou^2, 4^, Zhiguo Zhang^2, 4^, Jing Chen^2^, Wenke Feng^5^, Xiaokun Li^1^, Yi Tan^1, 2, 5 †^

*^1^ Chinese-American Research Institute for Diabetic Complications at the Wenzhou Medical University, Wenzhou, China;*

*^2^ Kosair Children's Hospital Research Institute, the Department of Pediatrics of the University of Louisville, School of Medicine, Louisville, USA;*

*^3^ Scool of Nursing, Wenzhou Medical University, Wenzhou, China;*

*^4^ Department of Cardiovascular Disorders of the First Hospital of Jilin University, Changchun, China;*

*^5^ Department of Pharmacology and Toxicology of the University of Louisville School of Medicine, Louisville, USA;*

***Running title****: FGF21 deletion aggravates diabetic cardiomyopathy*

***Word counts****: Abstract: 232*

*Main text/total: 4280/6703*

***Figures****: 7*

*† Corresponding author*

*Dr. Yi Tan:* *the Chinese-American Research Institute for Diabetic Complications at the Wenzhou Medical University, Wenzhou, China; or the Kosair Children's Hospital Research Institute, the Department of Pediatrics of the University of Louisville, School of Medicine, Louisville, KY;* [*y0tan002@louisville.edu*](mailto:l0cai001@louisville.edu)*.*

**Supplemental materials**

***Supplemental figures***

**Fig. S1.** STZ-induced type 1 diabetes decreased heart weight. At indicated time points after diabetes onset, heart weight changes are indicated by the ratio of heart weight to tibia length. Both WT and FGF21KO diabetic mice showed decrease in the heart weight since 1 m after diabetes onset compared to their respective controls. However, no significant differences in heart weight between WT diabetic and FGF21KO diabetic mice were observed. Data are presented as means ± SD (n≥5 for each group). * p<0.05 vs WT Ctrl group; # p<0.05 vs FGF21KO Ctrl group. Ctrl: control; DM: diabetes mellitus; WT: wild type; FGF21KO: FGF21 knockout; m: month(s).

**A**

**B**

**Fig. S2**. FGF21 deletion has no significant effects on blood pressure. At indicated time points after diabetes onset, blood pressure was measured using a CODA™ mouse/rat tail-cuff system. Both diastolic (A) and systolic (B) pressure showed no significant difference between WT and FGF21KO mice under diabetic and non-diabetic conditions. Data are presented as means ± SD (n≥5 for each group). Ctrl: control; DM: diabetes mellitus; WT: wild type; FGF21KO: FGF21 knockout; m: month(s).

***Supplemental tables***

**Table S1A****: Heart function**

(1 month after diabetes onset）

|  | WT/C  (n=5) | WT/DM  (n=5) | FGF21KO/C  (n=5) | FGF21KO/DM  (n=5) |
| --- | --- | --- | --- | --- |
| **IVS,d** | 0.63±0.02 | 0.62±0.01 | 0.63±0.01 | 0.62±0.01 |
| **LVID,d** | 3.79±0.07 | 3.65±0.06 **a** | 3.76±0.06 | 3.72±0.11 |
| **LVPW,d** | 0.79±0.04 | 0.62±0.04 **a** | 0.75±0.07 | 0.63±0.08 **b** |
| **IVS,s** | 1.1±0.07 | 1.07±0.03 | 1.12±0.05 | 1.13±0.04 |
| **LVID,s** | 1.98±0.04 | 1.93±0.02 | 1.95±0.03 | 1.95±0.05 |
| **LVPW,s** | 1.33±0.07 | 1.20±0.07 **a** | 1.35±0.08 | 1.16±0.07 **b** |
| **LV Vol,d** | 61.58±2.74 | 56.21±2.23 | 60.44±2.18 | 59.06±4.68 |
| **LV Vol,s** | 12.45±0.7 | 11.69±0.21 | 11.98±0.49 | 11.98±0.77 |
| **EF,%** | 79.81±0.45 | 79.32±1.03 | 80.19±0.44 | 79.68±1.11 |
| **FS,%** | 47.55±0.74 | 47.14±0.17 | 48.11±0.47 | 47.56±1.19 |
| **LV Mass** | 91.36±6.81 | 71.34±4.49 **a** | 87.64±8.45 | 75.63±9.82 |
| **LV Mass**  **Corrected** | 73.09±5.44 | 57.07±3.59 **a** | 70.11±6.76 | 60.2±8.99 |
| **Heart rate** | 456.2±75.3 | 407.8±70.7 | 475.4±36.6 | 422±58.7 |

**Table S1.** The parameters directly measured included left ventricle (LV) cavitary dimensions in diastole (LVID,d) and systole (LVID,s), LV posterior wall thickness in diastole (LVPW,d) and systole (LVPW,s), and interventricle septum thickness in diastole (IVS,d) and systole (IVS,s). LV fractional shortening (FS) %= [(LVIDd − LVIDs)/LVIDd] × 100; LV ejection fraction (EF) %= [(LV end-diastolic volume − LV end-systolic volume)/ LV end-diastolic volume] × 100. **a** *p*<0.05 vs. WT Ctrl group; **b** *p*<0.05 vs.FGF21KO Ctrl group. **c** *p*<0.05 vs. WT DM group. Ctrl: control; DM: diabetes mellitus; WT: wild type; FGF21KO: FGF21 knockout.

**Table S1B: Heart function**

(2 months after diabetes onset）

|  | WT/C  (n=5) | WT/DM  (n=5) | FGF21KO/C  (n=5) | FGF21KO/DM  (n=5) |
| --- | --- | --- | --- | --- |
| **IVS,d** | 0.63±0.01 | 0.62±0.01 | 0.63±0.01 | 0.63±0.01 |
| **LVID,d** | 3.75±0.07 | 3.62±0.09 | 3.71±0.06 | 3.63±0.13 |
| **LVPW,d** | 0.8±0.03 | 0.71±0.05 **a** | 0.76±0.04 | 0.69±0.06 |
| **IVS,s** | 1.09±0.02 | 1.06±0.03 | 1.10±0.03 | 1.04±0.04 **b** |
| **LVID,s** | 1.78±0.09 | 1.8±0.11 | 1.80±0.07 | 1.94±0.16 |
| **LVPW,s** | 1.47±0.02 | 1.34±0.06 **a** | 1.44±0.05 | 1.31±0.10 **b** |
| **LV Vol,d** | 59.9±2.62 | 55.09±3.09 | 58.58±2.06 | 55.79±4.82 |
| **LV Vol,s** | 9.6±1.16 | 9.75±1.56 | 9.81±0.93 | 11.94±2.53 |
| **EF,%** | 84.02±1.39 | 82.37±2.03 | 83.26±1.55 | 78.7±3.25 **b** |
| **FS,%** | 52.34±1.6 | 50.38±2.17 | 51.43±1.72 | 46.6±3.06 **b** |
| **LV Mass** | 90.85±5.12 | 77.71±6.99 **a** | 86.45±5.18 | 77.49±9.52 |
| **LV Mass**  **Corrected** | 72.68±4.09 | 62.17±5.59 **a** | 69.16±4.05 | 61.99±7.61 |
| **Heart rate** | 492.1±30.1 | 483.9±11.6 | 453.5±21.6 | 451.6±12.2 |

**Table S1C: Heart function**

(4 months after diabetes onset)

|  | WT/C  (n=5) | WT/DM  (n=7) | FGF21KO/C  (n=6) | FGF21KO/DM  (n=9) |
| --- | --- | --- | --- | --- |
| **IVS;d** | 0.62±0.00 | 0.61±.01 | 0.62±0.00 | 0.61±0.01 |
| **LVID;d** | 3.66±0.01 | 3.77±0.03 | 3.66±0.03 | 3.91±0.17 **b** |
| **LVPW;d** | 0.74±0.01 | 0.67±0.03 **a** | 0.74±0.01 | 0.62±0.04 **b,c** |
| **IVS;s** | 1.08±0.01 | 1.05±0.01 | 1.07±0.01 | 0.99±0.03 **b,c** |
| **LVID;s** | 1.83±0.03 | 2.22±0.05 **a** | 1.86±0.05 | 2.50±0.17 **b,c** |
| **LVPW;s** | 1.40±0.01 | 1.11±0.06 **a** | 1.36±0.02 | 0.99±0.04 **b,c** |
| **LV Vol;d** | 56.58±0.51 | 60.96±1.15 | 56.69±1.19 | 66.48±6.93 **b** |
| **LV Vol;s** | 10.15±0.50 | 16.65±0.86 **a** | 10.59±0.79 | 22.42±3.97 **b,c** |
| **%EF** | 82.06±0.98 | 72.68±1.52 **a** | 81.31±1.49 | 66.48±2.79 **b,c** |
| **% FS** | 50.00±1.06 | 41.11±1.31 **a** | 49.21±1.62 | 36.23±2.05 **b,c** |
| **LV Mass** | 81.32±1.05 | 79.19±3.42 | 81.65±1.83 | 80.58±9.15 |
| **LV Mass**  **Corrected** | 65.05±0.84 | 63.35±2.74 | 65.32±1.46 | 64.47±7.32 |
| **Heart rate** | 461.8±32.6 | 446.1±16.4 | 464.0±19.4 | 436.6±17.5 |
